# Supplementary material for: A Structural and Dynamic Analysis of the Partially Disordered Polymerase-Binding Domain in RSV Phosphoprotein
Source: Biomolecules. 2021 Aug 17;11(8):1225. doi: 10.3390/biom11081225 (PMC8392014; doi:10.3390/biom11081225)
Supplement: Supplementary file 1 [file biomolecules-11-01225-s001.zip › biomolecules-1319691-supplementary.pdf]

Supplementary

Table S1. NMR restraints and structural statistics for RSV $P_{127\text{-}163}$ tetramer.

**Number of structures**	20
**Number of NOE distance restrains per protomer**	629
Unambiguous	285
Intra-residue	133
Sequential	155
Medium range	56
Inter-molecular	166
Ambiguous	
Inter-molecular	166
**Number of dihedral angle restraints**	28Φ, 28Ψ
**Violation statistics**	
Distance violation/structure	
Unambiguous	
Viol > 0.5 Å	0.2 ± 0.9
Viol > 0.3 Å	10 ± 5
Ambiguous	
Viol > 0.5 Å	0.2 ± 0.9
Viol > 0.3 Å	6.0 ± 2.4
Dihedral angle violation/structure	
>5°	0
RMS of dihedral angle violation	0°
**RMS deviation from ideal covalent geometry**	
Number of close contacts	153
Bond angles	1.0°
Bond lengths	0.008 Å
**Rmsd**	
Backbone, all residues	1.39 Å
Heavy atoms all residues	1.73 Å
Backbone, ordered residues	0.4 Å
Heavy atoms, ordered residues	0.8 Å
**Ramachandran plot summary from Procheck**	
Most favored regions	99 %
Additionally allowed regions	1 %
Generously allowed regions	0 %
Disallowed regions	0 %
